# Plasmid- and strain-specific factors drive variation in ESBL-plasmid spread in vitro and in vivo

**DOI:** 10.1038/s41396-020-00819-4

**Published:** 2020-11-04

**Authors:** Fabienne Benz, Jana S. Huisman, Erik Bakkeren, Joana A. Herter, Tanja Stadler, Martin Ackermann, Médéric Diard, Adrian Egli, Alex R. Hall, Wolf-Dietrich Hardt, Sebastian Bonhoeffer

**Affiliations:** 1 Institute of Integrative Biology, Department of Environmental Systems Science, ETH Zurich, Zurich, Switzerland; 2 Swiss Institute of Bioinformatics, Basel, Switzerland; 3 Institute of Microbiology, Department of Biology, ETH Zurich, Zurich, Switzerland; 4 Department of Biosystems Science and Engineering, ETH Zurich, Basel, Switzerland; 5 Institute of Biogeochemistry and Pollutant Dynamics, Department of Environmental Systems Science, ETH Zurich, Zurich, Switzerland; 6 Department of Environmental Microbiology, Eawag, Duebendorf, Switzerland; 7 Biozentrum, University of Basel, Basel, Switzerland; 8 Division of Clinical Bacteriology and Mycology, University Hospital Basel, Basel, Switzerland; 9 Applied Microbiology Research, Department of Biomedicine, University of Basel, Basel, Switzerland

**Keywords:** Antibiotics, Microbial ecology, Phylogenomics

## Abstract

Horizontal gene transfer, mediated by conjugative plasmids, is a major driver of the global rise of antibiotic resistance. However, the relative contributions of factors that underlie the spread of plasmids and their roles in conjugation in vivo are unclear. To address this, we investigated the spread of clinical Extended Spectrum Beta-Lactamase (ESBL)-producing plasmids in the absence of antibiotics in vitro and in the mouse intestine. We hypothesised that plasmid properties would be the primary determinants of plasmid spread and that bacterial strain identity would also contribute. We found clinical *Escherichia coli* strains natively associated with ESBL-plasmids conjugated to three distinct *E. coli* strains and one *Salmonella* enterica serovar Typhimurium strain. Final transconjugant frequencies varied across plasmid, donor, and recipient combinations, with qualitative consistency when comparing transfer in vitro and in vivo in mice. In both environments, transconjugant frequencies for these natural strains and plasmids covaried with the presence/absence of transfer genes on ESBL-plasmids and were affected by plasmid incompatibility. By moving ESBL-plasmids out of their native hosts, we showed that donor and recipient strains also modulated transconjugant frequencies. This suggests that plasmid spread in the complex gut environment of animals and humans can be predicted based on in vitro testing and genetic data.

## Introduction

Plasmids can transfer horizontally between bacterial cells, within and between communities of the same or different species. They play a crucial role in bacterial ecology and evolution, because they often carry ecologically relevant accessory genes that allow bacterial populations to rapidly adapt to changing environments [1, 2]. Plasmids are also a major driver of antibiotic resistance evolution [3–5], and plasmid-encoded resistance determinants such as extended-spectrum beta-lactamases (ESBLs) have drawn particular attention [1, 6, 7]. Understanding the factors driving the spread of such plasmids is therefore important for our understanding of bacterial ecology and evolution, and for managing antibiotic resistance. This is particularly important in the mammalian gut, an environment that is a hot-spot of bacterial interaction [8, 9], where microbial communities consist of multiple species and frequently also diverse strains of the same species [10]. Despite important advances in recent decades, our ability to predict which plasmids will spread, and in which bacterial strains, in communities of enteric bacteria remains incomplete.

In vitro studies have revealed several key factors that drive changes in plasmid frequency over time. At the level of individual cells, plasmid incompatibility and surface- or entry exclusion can inhibit further plasmid acquisition [11, 12]. By contrast, some co-residing plasmids can enhance each other’s stability [13] and transfer [14, 15]. The host cell’s replication system [16] and other host properties also influence plasmid replication and stability [17]. Bacterial immunity systems such as CRISPR-Cas, or restriction modification systems (RM system) can eliminate incoming plasmids [18–21]. At the level of whole populations of bacteria, plasmid persistence depends on the frequency of plasmid loss during replication [17, 22], any growth costs associated with plasmid carriage [23, 24], and rates of horizontal acquisition [25]. Thus, both plasmid- and host-properties can influence plasmid stability in a cell and both a horizontal and a vertical component together can constitute the change in plasmid frequency over time, which we have defined as ‘plasmid spread’ for this study. Save for some notable exceptions [26, 27], plasmid spread has mostly been studied with plasmids not directly relevant for antibiotic resistance in nature/clinical settings, or with individual plasmids that have been introduced into well-defined model strains [11–13, 17, 18, 25, 28, 29]. Therefore, quantitative information about the relative contributions of plasmid- and host-determined properties to the spread of clinical plasmids from their native hosts would improve our overall understanding of horizontal gene transfer and bacterial evolution.

In addition to the biotic factors above, local abiotic conditions can play a key role in plasmid spread, potentially making it challenging to translate findings about plasmid dynamics in well-mixed in vitro conditions to natural or clinical settings. For example, some plasmids transfer more efficiently when bacteria are settled on surfaces, whereas others do so in well-mixed environments [30, 31]. Local environmental conditions, such as nutrient availability [27], can also impact the spread of mobile genetic elements by modulating the densities of interacting partners [2], influencing population structure [32], imposing physiological stress [33], or by modifying selection for plasmid-encoded traits [34]. Thus, plasmid spread in the natural environment of the mammalian gastrointestinal tract may differ from that observed in vitro. Most quantitative information about plasmid transfer and population dynamics comes from in vitro studies, whereas information about plasmids in the mammalian gut relies primarily on genomic studies [35–37], which lack direct observations of the dynamics and drivers of plasmid spread. Therefore direct, quantitative observations of plasmid spread in vivo, and comparison with classic types of in vitro experiments, would help translate findings from the laboratory back to nature. We, and others [38–41], have previously used mouse models to study processes that limit or boost plasmid spread in the gut [42–46]. These studies, however, were limited to laboratory strains and single conjugative plasmids without clinical relevance, with the exception of a single ESBL-plasmid used in [44]. To our knowledge, there are no studies that compare plasmids and their transfer dynamics quantitatively in vivo and in vitro.

Here, we use clinical *E. coli* strains and their natively associated ESBL-plasmids to test for variable plasmid spread among different bacteria/plasmid combinations, both in vitro and in mice. We combine these experimental data with bioinformatic analyses, identifying genetic determinants that influence plasmid spread in the absence of antibiotic selection. We chose to work with *E. coli* because some important commensal and pathogenic bacteria in the gastrointestinal tract come from the family Enterobacteriaceae, including some of the most important pandemic ESBL-producing strains [1, 6, 7, 47]. They often carry plasmids of the incompatibility groups IncF and IncI, which have low copy number and narrow host range [6, 48]. Our in vitro data showed the final frequencies of ESBL-plasmid-carrying recipient strains (transconjugants) varied greatly and were determined by plasmid, donor and recipient effects. As expected for conjugative plasmid transfer, the lack of the (*tra*) genes [49–51], which are well known to be required for conjugation, in three out of the eight clinical ESBL-plasmids tested, had the biggest impact on plasmid spread. Nevertheless, the donor and recipient strains also had a statistically significant effect on final transconjugant frequencies. Overall, our in vitro data qualitatively predicted plasmid spread in the antibiotic-free murine model for gut colonisation.

## Materials and methods

### Strains and growth conditions

We used eight ESBL-plasmid positive *E. coli* strains as plasmid donors (D1-D8). They were sampled from patients in a transmission study at the University Hospital Basel, Switzerland, and their ESBL-plasmids reflect relevant vectors of ESBL mediated drug resistance [52]. This collection comprised strains belonging to sequence types (ST) ST117, ST648, ST40, ST69, ST80, ST95, ST6697 and the very common ESBL sequence type ST131. We worked with 4 ESBL-plasmid negative recipient strains: RE1, a mouse-derived *E.coli* strain cured of its native IncI1 plasmid [45]; RE2 and RE3, two clinical *E. coli* isolates from healthy patients [52, 53]; and RS, the *Salmonella enterica* Typhimurium strain ATCC 14028 (RS). A comprehensive list of the plasmids found in these strains is given in Supplementary Table S1. Marker plasmids were introduced by electroporation, to mark recipients with either pACYC184 (New England Biolabs) encoding Chloramphenicol (Cm) resistance (except for RS, having chromosomal Cm resistance *marT::cat* [42]) or pBGS18 [54] encoding Kanamycin (Kan) resistance. Plasmid-borne resistance markers have their limitations, as they may interact with other plasmids present in these strains and potentially also affect their spread. Here, this effect seems minimal (Supplementary Fig. S1). Unless stated otherwise, we grew bacterial cultures at 37 °C and under agitation (180 rpm) in lysogenic broth (LB) medium, supplemented with appropriate amounts of antibiotics (none, 100 µg/mL Ampicillin (Amp), 25 µg/mL Cm in vitro and 15 µg/mL Cm prior to in vivo experiments, 50 µg/mL Kan). We stored isolates in 25% glycerol at −80 °C.

### Antibiotic resistance profiling

We used microdilution assays with a VITEK2 system (bioMérieux, France) to determine the minimum inhibitory concentrations (MIC). MIC breakpoints for ESBLs were interpreted according to EUCAST guidelines (v8.1). In addition, we confirmed resistance mechanism phenotypically, using ROSCO disk assays (ROSCO Diagnostica, Denmark), and/or genotypically with detection of CTX-M1 and CTX-M9 groups using the eazyplex Superbug assay (Amplex, Germany).

### In vitro conjugation experiment

To determine plasmid spread, which can include a vertical (clonal expansion) and a horizontal (conjugation) component, we calculated the final transconjugant frequency, which is the recipient population that obtained an ESBL-plasmid (transconjugants/(recipients+transconjugants), T/(R+T)), in a high throughput, 96-well plate-based assay. Donor and recipient populations grew overnight with or without Amp, respectively. We washed the independent overnight cultures by spinning down and resuspending and added ~1 µL of 6.5-fold diluted donor and recipient cultures into 150 µL fresh LB with a pin replicator (total ~1000-fold dilution, aiming to reach approximately a 1:1 ratio of donor and recipient). These mating populations grew for 24 h in the absence of antibiotics and were only shaken prior to hourly optical density (OD) measurements (Tecan NanoQuant Infinite M200 Pro). To determine the final cell densities, we plated the mating cultures at the end of the conjugation assay on selective LB-plates. In the first conjugation experiment, referred to as the 1st generation in vitro experiment, where the clinical strains transferred their native plasmids to recipients, we selected for donors+transconjugants with Amp, for recipients+transconjugants with Cm (*E. coli* recipients carried pACYC184-Cm and RS chromosomal *marT::cat*) and for transconjugants with Amp+Cm. For a second, separate conjugation experiment, referred to as the 2nd generation in vitro experiment, we chose a subset of transconjugants generated in the 1st generation in vitro experiment as new plasmid donors. Transconjugants isolated from the 1st generation experiment were frozen and regrown before the 2nd generation experiment. The resulting culturing steps make differences between the 1st and 2nd generation experiments due to transient plasmid de-repression in transconjugants unlikely [55, 56]. Transconjugants and recipients of the clone type RE3 were omitted because of the size of the experiment, and transconjugant RE2 carrying p1B_IncI had to be excluded as plasmid donor due to insufficient freezer stocks. We selected for donors with Cm, for recipients with Kan (recipients carried pBGS18-Kan) and for transconjugants with Kan+Amp. A single transconjugant colony would be equivalent to 20 CFU/mL, which approximates our detection limit. We performed experiments with *E. coli* recipients and *S*. *Typhimurium* recipient RS as independent experiments and the 1st generation in vitro (*n* = 4–6) and 2nd generation in vitro (*n* = 6) experiments each in two replica blocks.

The plasmids in our conjugation experiments could either be transferred in the liquid growth phase or after plating on selective plates (surface mating). To assess the extent of surface mating we performed an additional experiment, where we treated donors and recipients as above but grew them in separate liquid cultures, instead of mixed cultures, only mixing them immediately before plating on selective LB-plates. To make a direct comparison between some of the donor-plasmid-recipient combinations used in the 1st generation and 2nd generation in vitro experiments, we performed a conjugation experiment as described above with D1 (with and without pACYC184) and transconjugants RE1 and RS carrying plasmid 1B_IncI, isolated from the 1st generation in vitro experiment, as plasmid donors.

### **In vitro** plasmid cost experiment and other growth rate measurements

To investigate the effect of ESBL-plasmid carriage on bacterial growth in absence of antibiotics, we measured the growth rate of transconjugants and recipients. Per donor and recipient combination, we used three transconjugants, four replicates each, obtained from independent mating populations of the 1st generation in vitro experiment. Transconjugants for which we did not store three independent transconjugants were excluded from this analysis. We grew bacterial cultures in the absence of antibiotics overnight and diluted them 150-fold by transfer with a pin replicator to a 96-well plate, containing 150 µL fresh LB per well. We incubated the cultures without shaking and estimated growth rates of recipients and transconjugants based on ten manual OD measurements over 24 h. We estimated growth rates (h^−1^) using the R package Growthcurver [57]. We expressed plasmid cost as the growth rate of transconjugants relative to the corresponding ESBL-plasmid free recipient. Transconjugants have experienced longer growth under laboratory conditions than recipients (conjugation experiment). To verify that this did not affect our estimates of plasmid cost, we conducted a third growth rate experiment. Prior to this experiment, recipients were grown under the same conditions as in the conjugation experiment but without donor strains. We then measured and compared the growth rates of these strains and the original recipients that had not undergone the additional culturing step (Supplementary Fig. S2).

Other growth rate measurements (Supplementary Figs. S2, S3) were performed as follows: We grew bacterial cultures with appropriate antibiotics overnight, washed and diluted them ~1000-fold. For the growth measurements, bacteria grew in the absence of antibiotics and the plate reader measured OD every hour for 24 h. Again we estimated growth rates (h^−1^) using the R package Growthcurver [57].

### **In vivo** experiments

We have previously established a murine model for enterobacterial pathogen infection [58] that allows the tracking of plasmid dynamics [42–46]. For conjugation experiments, we used 8–16 week old C57BL/6 mice that contain an oligo microbiota allowing colonisation of approximately 10^8^ *E. coli* per gram faeces [59]. *E. coli* stool densities of up to 10^8^ CFU/g have also been detected in healthy human volunteers [53]. We infected 7–10 mice per treatment group (minimum of two independent experiments; no antibiotic pre-treatment) orogastrically with ~5 × 10^7^ CFU of RE2 or RE3, carrying marker plasmid pACYC184 and 24 h later with ~5 × 10^7^ CFU of either D4, D7 or D8. Faeces were collected daily, homogenised in 1 mL of PBS with a steel ball by a Tissue Lyser (Qiagen) at 25 Hz for 1 min. We enumerated bacterial populations by selective plating on MacConkey media (selection for donors+transconjugants with Amp (100 µg/mL), for recipients+transconjugants with Cm (15 µg/mL) and for transconjugants with Amp+Cm) and calculated final transconjugant frequencies T/(R+T).

For competition experiments we infected 8–16-week-old C57BL/6 oligo microbiota mice orogastrically with a 1:1 mixture of both competitor strains (~5 × 10^7^ CFU total; no antibiotic pre-treatment). We collected faeces and enumerated bacterial populations daily. We plated bacteria on MacConkey agar containing Cm and replica-plated on media containing Cm, Kan, and Amp to select the transfer deficient transconjugants. A change in fitness conferred by plasmid carriage is reflected in the relative frequency of recipients to transconjugants (R/T).

Prior to all infections, we subcultured the overnight cultures (LB containing the appropriate antibiotics) for 4 h at 37 °C without antibiotics (1:20 dilution) to ensure equal densities of bacteria. Cells were washed in PBS and introduced into mice. All infection experiments were approved by the responsible authority (Tierversuchskommission, Kantonales Veterinäramt Zürich, license 193/2016 and license 158/2019).

### Sequencing, assembly, annotation

We sequenced all donor and recipient strains with Illumina MiSeq (paired end, 2 × 250 bp), Oxford Nanopore MinION and PacBio Sequel methods. We produced hybrid assemblies with Unicycler [60] (v0.4.7) and used the most contiguous assemblies (Oxford Nanopore—Illumina for D1, D2, D4, D6, D7, D8 and Pacbio Sequel—Illumina for D3, D5, RE1, RE2, RE3). Manual curation involved removing contigs smaller than 1kB, and sequences up to 5 kB that mapped to the own chromosome. We performed quality control by mapping the paired end Illumina reads to the finished assemblies using samtools (v1.2) and bcftools (v1.7) [61, 62]. For recipient RS, the ancestral strain was sequenced with Illumina (2 × 150 bp), and mapped against the reference sequence, downloaded from NCBI Genbank under the accession numbers NZ_CP034230.1 and NZ_CP034231.1.

To study the genetic contribution to the observed variation in plasmid spread, we sequenced various transconjugants from the 1st and 2nd generation in vitro experiments as well as the in vivo transfer experiment (Supplementary Table S2). In vitro: three clones from independent mating populations for RE3 carrying plasmid p4A_IncI or p8A_IncF and one clone for the other transconjugants. In vivo: eight clones of RE3 carrying p4A_IncI isolated from five mice on day 7 post donor infection, and eight clones of RE3 carrying p8A_IncF isolated from six mice on day 2 (1 clone), day 6 (3 clones), or day 7 (4 clones) post donor infection. Resequencing was performed on an Illumina MiSeq (paired end, 2 × 150 bp) and we mapped the reads to the closed assemblies of respective donor and recipient strains using the breseq pipeline (v 0.32.0) [63]. Mutations or indels shared by all re-sequenced strains were treated as ancestral (Supplementary Table S2).

To investigate the transfer of plasmid p8C_IncBOKZ, we screened 3–5 transconjugants from independent mating populations per conjugation pair (Supplementary Table S2). We performed PCRs with primers specific to IncB/O and IncK plasmids [64], (5′ to 3′): MRxeBO_K_for: GAATGCCATTATTCCGCACAA and MRxeBO_K _rev; GTGATATACAGACCAT-CACTGG).

To extract a chromosomal alignment of the *E. coli* donor and recipient strains, we concatenated the genes returned by core genome Multi-Locus Sequence Typing (cgMLST) for all strains. We used the chewBBACA software to type these strains according to the Enterobase cgMLST scheme [65, 66]. We inferred the phylogenetic tree using BEAST2 [67], with an HKY substitution model, a tight prior on the mutation rate (constrained around the *E. coli* mutation rate of 10^−4^ mutations per genome per generation, as estimated by Wielgoss et al. [68], and assuming 100–10,000 generations per year), and a birth–death tree prior (priors are listed in Supplementary Table S3). The timing of the tree was additionally informed by the sampling dates of the strains: For clinical donor strains this corresponded to the isolation date in the hospital; recipient RE1 was isolated from a mouse co-infected with Salmonella on 17.06.2007; and for recipients RE2 and RE3 we assumed the start of the 3-month study conducted by Wotzka et al. [53], i.e. 30.01.2015. We performed bacterial genome annotation using Prokka [69], and determined the sequence type (ST) using mlst (Torsten Seemann, https://github.com/tseemann/mlst), which makes use of the PubMLST website (https://pubmlst.org/) developed by Keith Jolley [70]. Phylogroups were assigned using ClermonTyper [71].

We determined genomic features using a range of bioinformatic tools, and by BLAST comparison against various curated databases. Plasmid replicons and resistance genes were identified using abricate (Torsten Seemann, https://github.com/tseemann/abricate) with the PlasmidFinder [72] and ResFinder [73] databases respectively. We located phages using PHASTER [74] (listing only those marked as ‘complete’), type 6 secretion systems using SecReT6 [75], virulence genes using the Virulence finder database [76], toxin–antitoxin (TA) systems using the database TADB 2.0 [77], and CRISPR-Cas loci using CRISPRCasFinder [78]. We found restriction-modification (RM) systems using the grep function on the term ‘restriction’ in the general feature format files from prokka, and verified them with the RM-database Rebase [79]. To determine the presence/absence of IncF and IncI transfer genes, we constructed our own database as a reference. IncF transfer genes were taken from the supplementary material of Fernandez-Lopez et al. [80], IncI1 transfer genes from plasmids R64 using the annotations by Komano et al. [49], and IncIγ transfer genes from the plasmid R621a annotated by Takahashi et al. [50].

### Construction of non-transferrable plasmids

For the in vivo competition experiments, we generated non transferrable plasmids for three independent transconjugants. We deleted their origin of transfer (*oriT*) region using the lambda red recombinase system with pKD4 as template for the Kan resistance marker [81]. The following primers were used (5′ to 3′): For IncI plasmids (p4A_IncI) DIncI_oriTnikA_f (GCATAAGACTATGATGCACAAAAATAAC-AGGCTATAATGGGTGTAGGCTGGAGCTGCTTC) and DIncI_oriTnikA_r (CCTTCTCTTTTTCG-GAATGACTGCATTCACCGGAGAATCCATGGGAATTAGCCATGGTCC) [45] and for F plasmids (p8A_IncF) D25_2_oriT-nikA-ko_vw (CCATGATATCGCTCTCAGTAAATCCGGGTCTATTTTGTA-AGTGTAGGCTGGAGCTGCTTC) and D25_2_oriT-nikA-ko-rev (GTGCGGACACAGACTGGATATTT-TGCGGATAAAATAATTTATGGG-AATTAGCCATGGTCC). We verified all mutants by PCR (IncI1_oriT _val_f: AGTTCCTCA-TCGGTCATGTC, IncI1_oriT_val_r: GAAGCCATTGGCACTTTCTC, D25_oriT_val_fw: CATACAGG-GATCTGTTGTC and D25_2_oriT_ver_rv: CAGAATCACTAT-TCTGACAC) and experimentally by loss of transfer function.

### Statistical analyses

For in vitro experiments we performed analyses using R (version 3.4.2). The effects of donor, recipient and plasmid on final transconjugant frequency were analysed with either a two-way ANOVA (1st generation in vitro experiment with factors donor-plasmid pair and recipient) or a three-way ANOVA (2nd generation in vitro experiment with factors donor, plasmid, recipient). For the 1st generation in vitro experiment, we excluded strain–plasmid pairs which did not result in transconjugants (D2, D3, D7) and recipient RS from this analysis. When single replicates for a given donor–recipient combination lacked transconjugants (D5 and D6), we assigned these replicates a final transconjugant frequency at the detection limit of 10^−8^. The data of the 2nd generation in vitro experiment were not fully factorial. To enable testing of interactions, we therefore performed two 3-way ANOVAs: one excluding plasmid p1B_IncI and one excluding donor RE2, for which we had to take the two replicate blocks into account: *P* < 0.001). For two replicate populations (RS self–self transfer with p1B_IncI), we had higher counts on plates selecting for transconjugants than on plates selecting for recipients+transconjugants and replaced the resulting negative CFU/mL for recipients with 0 CFU/mL (we assume the higher count on selective agar reflects measurement error, given the true frequency of plasmid-carrying cells cannot exceed 1.0).

For statistical comparisons derived from in vivo experiments, Kruskal–Wallis tests were performed with Dunn’s multiple test correction using GraphPad Prism Version 8 for Windows.

## Results

### Strains and plasmids

**Fig. 1:**
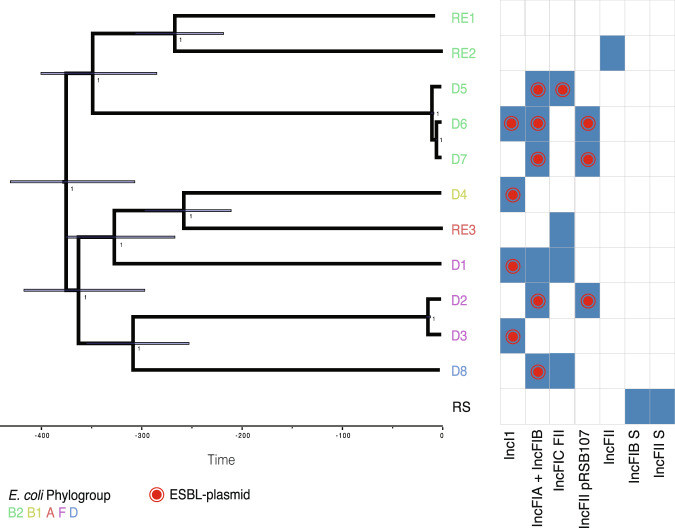
Phylogenetic tree of the *E. coli* donor and recipient strains, inferred using Bayesian inference on a core genome alignment. Strain names at the tips are coloured by *E. coli* phylogroup. The *S. Typhimurium* recipient RS was not included in the phylogeny, but listed here to allow comparison of the plasmid content. RE1–3 denotes the three *E. coli* recipients and D1–8 denotes the eight donors. Bars on internal nodes indicate the 95% highest posterior density interval of the node age in years, numbers indicate the posterior probability for a given bifurcation. Blue rectangles show that a plasmid with the indicated IncF or IncI replicon (incompatibility marker) is present in that strain. A red dot indicates the replicon(s) present on the ESBL-plasmid in each strain. Coloured letters indicate the *E. coli* phylogroup.

As potential ESBL-plasmid donors we used eight clinical *E. coli* strains (D1-D8), which were selected at the University Hospital Basel to be representative for the clinically relevant diversity of ESBL-plasmid positive strains. We chose four recipient strains susceptible to β-Lactam antibiotics, of which three are *E. coli* (RE1-RE3) and one *S*. Typhimurium (RS, Supplementary Fig. S4, Supplementary Table S4). Sequence analysis revealed a large phylogenetic diversity, with donor strains belonging to phylogenetic groups B1, B2, D or its subgroup F, and recipients to either B2 or A (Fig. 1). We also observed diverse accessory traits such as bacterial immunity systems (Supplementary Fig. S5) and virulence genes (Supplementary Fig. S6). All but two strains encode type 6 secretion systems (T6SS), and strain D4 shows an enteropathogenic virulence profile. Each strain carries at least one and up to eight plasmids of various incompatibility groups (Supplementary Fig. S7, Table S1). Every donor strain harbours a single antibiotic-resistance plasmid (the ESBL-plasmid), either of the plasmid family IncI or IncF (Table 1) and displayed an ESBL-resistance phenotype (Supplementary Table S4). All strains encode numerous intact prophage sequences in their chromosome (Supplementary Fig. S8) and we found P1-like phages, i.e. prophages that move like plasmids in their lysogenic phase [82, 83], in various strains (Supplementary Fig. S9). ESBL-plasmid p2A_IncF carries a SPbeta-like prophage (68.4 kB), which encodes all 12 resistance genes of that plasmid. With the exception of p3A_crypt and pRE3B_crypt, all plasmids bigger than 35 kB carry plasmid addiction systems [22] (TA systems, Supplementary Fig. S10).

**Table 1:** Clinical ESBL-plasmids in their native bacterial host strains (donors). Plasmids names contain an abbreviation for the original host strain, i.e.1 for D1, a letter allowing to separate the different plasmids within this host strain and the plasmid replicon type.

Strain (*E. coli* sequence type)	ESBL-plasmid	Plasmid size	Plasmid replicon (incompatibility group)	Resistance genes on the ESBL-plasmid^a^	Antibiotic resistance phenotype^b^
D1 (ST 117)	p1B_IncI	111 kB	IncIγ	aadA5; ***bla***_**CTX-M-1**_; dfrA17; sul2	Amp, Ceftri, Cotri
D2 (ST 648)	p2A_IncF	165.7 kB	IncFIA; IncFIB; IncFII	mph(A); catB3; aadA5; aac(6′)-Ib-cr; dfrA17; sul1; sul2; ***bla***_**OXA-1**_; tet(A); ***bla***_**CTX-M-27**_; aph(3″)-Ib; erm(B)	Amp, Amo/C; Ceftri; Tobra; Cotri; Cipro
D3 (ST 648)	p3B_IncI	59.8 kB	IncI1	***bla***_**CMY-42**_	Amp; Amo/C; Pip-T; Ceftaz; Ceftri; Tobra; Amika; Cotri; Cipro
D4 (ST 40)	p4A_IncI	88.9 kB	IncIγ	***bla***_**CTX-M-1**_	Amp; Ceftri
D5 (ST 131)	p5A_IncF	160 kB	IncFIB; IncFIC(FII); IncFIA	mph(A); aadA5; aac(6′)-Ib-cr; dfrA17; sul1; ***bla***_**CTX-M-15**_; aac(3)-IIa; tet(A); catB3; ***bla***_**OXA-1**_	Amp; Amo/C; Ceftaz; Ceftri; Cefe; Tobra; Cotri; Cipro
D6 (ST 131)	p6A_IncI/F	157 kB	IncI1; IncFIA; IncFIB; IncFII	***bla***_**TEM-1B**_, aac(3)-IId, ***bla***_**CTX-M-8**_	Amp; Ceftri; Cefe; Tobra; Cipro
D7 (ST 131)	p7A_IncF	134.9 kB	IncFIA; IncFIB; IncFII; Col156	mph(A); aph(3″)-Ib; aadA5; dfrA17; sul1; sul2; tet(A); ***bla***_**CTX-M-27**_	Amp; Ceftri; Cotri; Cipro
D8 (ST 69)	p8A_IncF	131 kB	IncFIA; IncFIB	dfrA14, mph(A), ***bla***_**CTX-M-14**_, tet(B)	Amp; Ceftri; Cotri

^a^Genes encoding beta-lactamases are highlighted in bold.

^b^Antibiotic resistance phenotype is defined as being above the EUCAST defined minimum inhibitory concentration breakpoint (MIC; see S4 Table for all antibiotics tested and MIC information). Donor strains fulfilled criteria for ESBL production based on EUCAST recommendations. ESBL phenotypes were confirmed.

*Amp* ampicillin; *Amo/C* amoxicillin/clavulanic acid; *Pip-T* piperacillin-tazobactam; *Ceftaz* ceftazidim; *Ceftri* ceftriaxone; *Cefe* cefepim; *Tobra* tobramycin; *Amika* amikacin; *Cotri* cotrimoxazol; *Cipro* ciprofloxacin.

### Plasmid spread in the absence of antibiotics varies depending on the clinical donor and the recipient strain

**Fig. 2:**
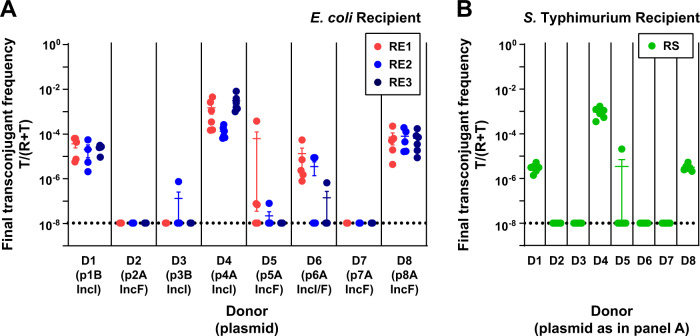
ESBL-plasmids spread at variable rates in the absence of antibiotics (1st generation in vitro experiment). Plasmid spread was measured as the final transconjugant frequency, i.e. the ratio of the recipient population carrying the ESBL-plasmid (T), relative to the total of plasmid-free (R) and plasmid carrying (T) recipient populations. Final transconjugant frequency is shown for recipient populations of *E. coli* strains RE1-3 (A) and *S. Typhimurium* strain RS (**B**). Circles represent independent replicates (*n* = 4–6) and the beams are mean values ± standard error of the mean (SEM). The detection limit was at ~10^−8^. Total population densities can be found in Supplementary Fig. S11.

To test whether the spread of ESBL-plasmids in recipient populations varied depending on the identity of donor (clinical isolates, each with different ESBL-plasmids) and recipient bacteria, we first performed conjugation experiments with all possible donor–recipient combinations in the absence of antibiotics (referred to as 1st generation in vitro experiment). We used the final transconjugant frequency, i.e. the fraction of the recipient population that carried the ESBL-plasmid after 24 h, to measure plasmid spread. The highest final transconjugant frequency (~0.1%) was achieved when plasmid p4A_IncI spread in populations of recipient RE3. Five of the eight ESBL-plasmids transferred at detectable levels to more than one of the *E. coli* recipients and their final transconjugant frequencies spanned five orders of magnitude (Fig. 2A, Supplementary Fig. S11). The average final transconjugant frequency varied depending on the donor–plasmid pair and among recipient strains (two-way ANOVA excluding D2, D3, D7 with non-conjugative ESBL-plasmids, effect of donor–plasmid pair: *F*_4,66_ = 87.665*, P* < 0.01, effect of recipient: *F*_2,66_ = 5.439, *P* < 0.01). The variation among donor–plasmid pairs depended also on the recipient (donor-plasmid pair × recipient interaction: *F*_8,66_ = 3.164, *P* < 0.01). Although these ESBL-plasmids are natively associated with *E. coli*, they reached comparable maximal transconjugant frequencies in the RS (*S*. *Typhimurium*) recipient populations (Fig. 2B). With RS, variation across donor–plasmid pairs was similar to that obtained with *E. coli* recipients, with the exception of p6A_IncI, which did not transfer to recipient RS.

The pili of type IncI and IncF plasmids support plasmid transfer on solid surface and in liquid growth environment [30, 84]. The protocol of our in vitro experiment potentially allowed for both liquid- and surface- (after plating) mating. To determine whether surface mating contributed significantly to transfer among these strains in these conditions, we performed a separate experiment which only allows for surface mating, for a subset of donor–recipient combinations (Supplementary Fig. S12). In this experiment, only p1B_IncI and p4A_IncI transferred from donor to recipient, resulting in transconjugant frequencies ranging from 10^−8^ to 10^−5^, in a recipient-dependent manner (two-way ANOVA excluding D6, D8 and RS: effect of recipient: *F*_2,20_ = 34.29, effect of donor–plasmid pair: *F*_1,20_ = 12.88, *P* < 0.01 in both cases). This suggests that plasmid transfer in our 1st generation in vitro experiments took place primarily during the 24-h liquid-growth phase, although depending on the plasmid there can also be additional after-plating conjugation.

### Plasmid, donor and recipient lead to variation in plasmid spread

**Fig. 3:**
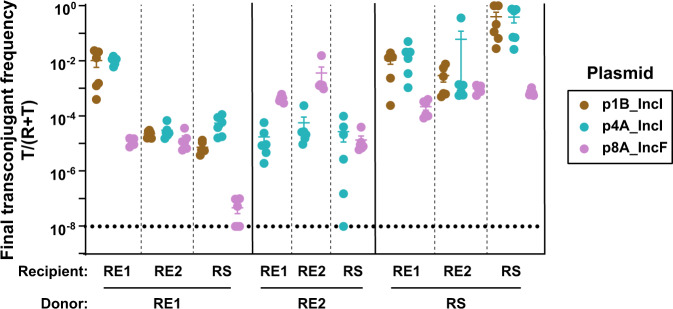
Final transconjugant frequency depends on donor, recipient, and plasmid (2nd generation in vitro experiment). Eight transconjugants isolated from mating assays in the 1st generation in vitro experiment (Fig. 2), used here as plasmid donor strains, transferred their plasmid to three different recipients. Circles represent independent replicates (*n* = 6), the beams are mean values ± SEM and different plasmids are indicated in colour. The detection limit was at ~10^−8^. Total population densities can be found in Supplementary Fig. S13. Donor RE2 carrying plasmid p1B_IncI was excluded, see ‘Methods'.

In the 1st generation in vitro experiment (Fig. 2), where we investigated the spread of ESBL-plasmids from their native hosts, each plasmid was present in a single donor. Therefore, we could not separate the contributions of plasmid and donor strain to the observed variation of plasmid spread. To do so, we performed a second conjugation experiment (referred to as the 2nd generation in vitro experiment) with plasmids that showed notable plasmid transfer in the 1st generation in vitro experiment. We held conditions identical to the 1st generation in vitro experiment, but in the 2nd generation in vitro experiment each donor strain background carried one of several ESBL-plasmids and each ESBL-plasmid was represented in multiple donor strains. Specifically, we used eight transconjugants isolated from the 1st generation in vitro experiment as plasmid donors and three of the same recipient strains (Fig. 3, Supplementary Fig. S13). The final transconjugant frequency varied among donor strains and among plasmids (three-way ANOVA with plasmid, excluding p1B_IncI, donor and recipient as factors, effect of donor strain: *F*_2,90_ = 150.133, *P* < 0.001, effect of plasmid: *F*_1,90_ = 49.717, *P* < 0.001). Variation among plasmids depended on both, the recipient and the donor strain (donor strain × plasmid interaction: *F*_2,90_ = 96.352, *P* < 0.001; recipient × plasmid interaction: *F*_2,90_ = 29.610, *P* < 0.001). For instance, when the donor and recipient strains were both RS, both IncI ESBL-plasmids yielded remarkably high final transconjugant frequencies of 40%. A second analysis supported variation among donor strains and plasmids and that variation among plasmids depended on recipient and donor strain (three-way ANOVA excluding RE2, effect of donor strain: *F*_1,93_ = 560.269, *P* < 0.001, effect of plasmid *F*_2,93_ = 156.075, *P* < 0.001, recipient × plasmid interaction: *F*_4,93_ = 26.104, *P* < 0.001, donor strain × plasmid interaction: *F*_2,93_ = 3.999, *P* = 0.022). As in our 1st generation in vitro experiment, average final transconjugant frequencies also varied among recipients (*P* < 0.001 for effect of recipient in both three-way ANOVAs). Thus, the final frequency of transconjugants depended on donor strain, plasmid, and recipient. Also, we found relatively high final transconjugant frequencies when donor and recipient were plasmid+/plasmid- versions of the same strain (Fig. 3, self–self transfer).

For some plasmid–recipient combinations, we noticed that replacing the native donor strains with transconjugants from the 1st generation in vitro assay (primary and secondary plasmid transfer, respectively) led to large differences in final transconjugant frequencies (Figs. 2 and 3). For a subset of strains, we tested whether these resulted solely from the substitution of the native donor strain (D1) with a secondary donor strain (RE1 or RS, Supplementary Fig. S1). When RE1 or RS acted as donor strain for plasmid p1B_IncI, transconjugant frequencies of RE1 carrying p1B_IncI increased 45-fold and 112-fold, respectively, compared to when p1B_IncI was transferred from its native donor strain D1. When both donor and recipient were RS, the final transconjugant frequency increased 2800-fold compared to transfer of plasmid p1B_IncI from its native host D1 to RS. This shows plasmid transfer from a secondary bacterial host can differ strongly from its transfer from the initial host.

### ESBL-plasmids can spread rapidly in vivo, with efficiencies corresponding to in vitro trends

**Fig. 4:**
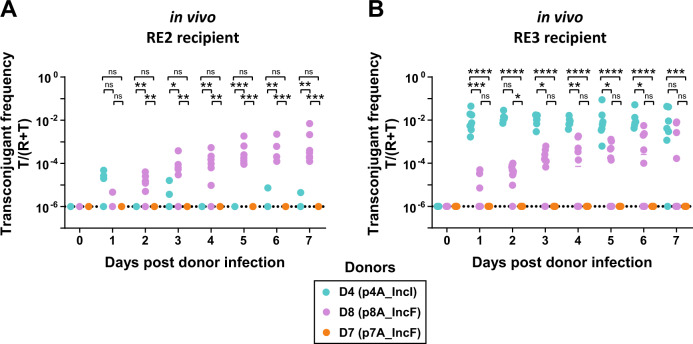
ESBL-plasmids can spread in the gut in the absence of antibiotic selection. We measured the spread of three plasmids as final transconjugant frequency in two distinct recipient populations, **A** RE2 and **B** RE3, and enumerated transconjugants in faeces by selective plating. Dotted lines indicate the detection limit for selective plating. Circles represent independent replicates (*n* = 7 for RE2 conjugations; *n* = 7 for D4-RE3; *n* = 10 for D8-RE3 and D7-RE3), lines show the median and different donor-plasmid pairs are indicated in colour. Kruskal–Wallis test *p* > 0.05 (ns), *p* < 0.05 (*), *p* < 0.01 (**), *p* < 0.001 (***), *p* < 0.0001 (****). Total population densities can be found in Supplementary Fig. S14.

To test the effect of plasmid-donor pair and recipient strain on plasmid spread in a complex environment, we performed conjugation experiments in gnotobiotic mice with a defined multispecies microbiota [59] over 7 days and in the absence of antibiotics. This resident microbiota allows colonisation of ~10^8^ *E. coli* per gram faeces, *E. coli* densities representative of the guts of some humans and animals [10, 53]. We used three clinical donors (D4, D8 and D7), and two recipients (RE2 and RE3, Fig. 4), a subset of strains that reflects diversity in plasmid type (that is, incompatibility group and transfer efficiency in vitro; Figs. 1, 2), and in genetic properties of the strain (that is, variable virulence factors, phages or other accessory genes; Fig. 1, Supplementary Figs. S6, S8, S5).

The variation of ESBL-plasmid spread in vivo was in qualitative agreement with the 1st generation in vitro experiment. As in the 1st generation in vitro experiment (Fig. 2) for recipient RE3 we observed highest transconjugant frequencies with plasmid p4A_IncI followed by p8A_IncF and no transconjugants with p7A_IncF (Fig. 4, Supplementary Fig. S14). Furthermore, the final transconjugant frequency with plasmid p4A_IncI was higher with RE3 than RE2 both in vivo and in vitro (see Fig. 4A,B, blue dots, and Fig. 2). Lastly, the final transconjugant frequencies with p8A_IncF were similar for both recipient populations (Fig. 4A,B, purple dots, and Fig. 2). Because the final frequency of RE3 carrying p4A_IncI (1%) was already reached at day 1, we re-performed this conjugation experiment, sampling more densely in time and found the final transconjugant frequency to be established already 8 h after the orogastric introduction of donor D4 (Supplementary Fig. S15). This rapid increase in transconjugant frequency was followed by a 6-day plateau, which may result from the simultaneous decrease of recipient and transconjugant populations over time (Supplementary Fig. S14). Indeed, direct competition experiments in vivo (Supplementary Fig. S16) confirmed the competitive advantage of donor D4 over RE3. This fitness benefit may be explained by the difference in growth rate, as estimated in vitro (Supplementary Fig. S3).

In vitro, we found that plasmid spread from donor strains and from transconjugants to recipient strains could vary by several orders of magnitude (Supplementary Fig. S1). Such differences in plasmid spread from primary and secondary donor could also be present in vivo. However, with the exception of RE3 carrying p4A_IncI, the transconjugant populations were minor compared to the size of the donor populations throughout the in vivo experiment (Supplementary Fig. S14). Thus, for plasmid spread to be dominated by transfer from transconjugants, plasmid transfer rates from transconjugants would need to be 10^4^-fold higher than transfer rates from the donor strain.

### No cost of ESBL-plasmid carriage detected and variable horizontal plasmid transfer probably drives variation of plasmid spread in vitro and in vivo

**Fig. 5:**
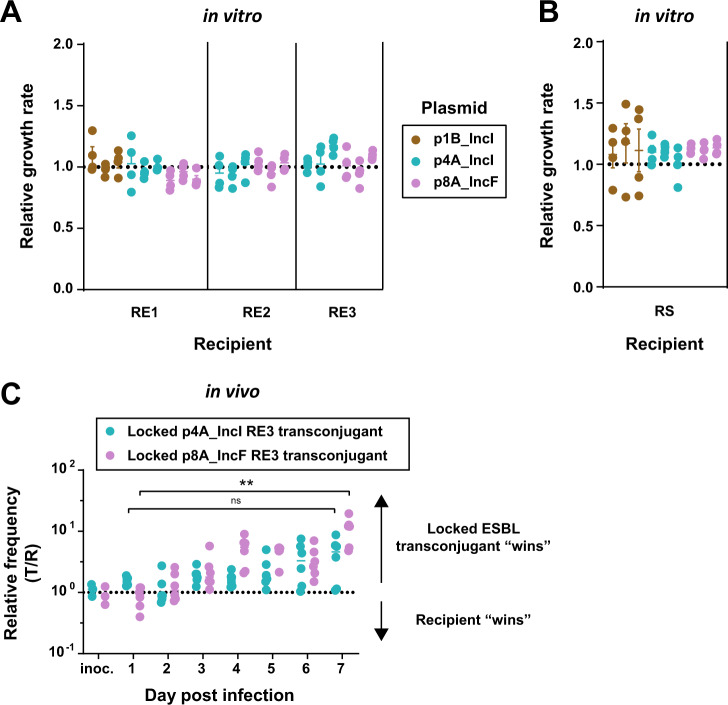
No evidence for cost of plasmid carriage for transconjugants. **A**, **B** We measured plasmid cost for ten strain-plasmid combinations, with three independently isolated transconjugants each (*n* = 4; beams are mean values ± SEM). Transconjugants and their plasmid free complements grew in independent cultures and we calculated the relative growth by dividing the transconjugant growth rates (h^−1^) by the mean growth-rate of plasmid-free strains. **C** We performed the competition experiment by colonising the mice with a 1:1 mix of a non-conjugative transconjugant (oriT-knockout) and recipient RE3 (*n* = 6; 3 independent transconjugants, *n* = 2 for each). **C** Kruskal–Wallis test *p* > 0.05 (ns), *p* < 0.01 (**).

The observed variation of final transconjugant frequencies in vitro and the plasmid dynamics in the mouse gut could potentially be explained by variable rates of horizontal plasmid transfer, or by variable rates of clonal expansion of transconjugants (driven by variable effects of the plasmid on bacterial growth depending on the plasmid or recipient strain). To investigate this, we first tested whether these plasmids were associated with growth costs for ten strain-plasmid combinations in vitro. We estimated plasmid growth cost as the growth rate of transconjugants relative to their respective plasmid-free recipient strain, and in this experiment, we found no significant effect of plasmid carriage on bacterial growth rates for the tested strains (Fig. 5A,B, Student’s *t* test for *E. coli* hosts and Wilcoxon Rank Sum Test for *S*. *Typhimurium*, *P* > 0.05 in all cases, before and after Holm’s correction for multiple testing). This suggests variable rates of clonal expansion of transconjugants relative to recipients are unlikely to explain the variation in final transconjugant frequencies we observed (Fig. 2). Consistent with this, we found no correlation between average final transconjugant frequencies (for the combinations tested in the experiment in Fig. 2A) and average growth rate difference between transconjugants and recipients (Pearson *r*^2^ = 0.58, *P* = 0.18).

An alternative explanation for the observed variation of final transconjugant frequencies across different plasmid/bacteria combinations is variation of horizontal transfer rates. To verify that horizontal transfer contributed substantially to observed final transconjugant frequencies, we calculated whether clonal expansion of transconjugants, after a single transfer event, would have been sufficient to explain observed final transconjugant frequencies in our in vitro assay (Fig. 2). This was only the case for *S*. *Typhimurium* recipients carrying p1B_IncI and p8A_IncF, which showed a consistent trend towards higher growth rates relative to the ESBL-plasmid-free *S*. Typhimurium recipient (Fig. 5B), but not for any plasmid in any *E. coli* recipient population (Supplementary Results). This indicates there was appreciable horizontal transfer (multiple events) in the majority of combinations we tested. A third process that potentially contributes to variable transconjugant frequencies is segregational plasmid loss. We did not test this directly, but note that each ESBL-plasmid encodes at least two TA-systems (Supplementary Fig. S10). We would expect these to make plasmid loss from transconjugants infrequent [22].

In vivo, we investigated the effect of ESBL-plasmids p4A_IncI and p8A_IncF on bacterial fitness with direct 1:1 competition between recipient RE3 and its transconjugants (Fig. 5C). After 7 days of competition, for transconjugants with p4A_IncI, there was no significant change in the relative frequency of recipients to transconjugants. The in vivo competition experiment revealed a growth advantage of RE3 when carrying p8A_IncF, allowing a tenfold relative increase of the initial transconjugant frequency when growing for 7 days in the gut (from a 1:1 ratio to a 1:10 ratio of recipients to transconjugants, Fig. 5C). Because we used *oriT*-mutants, no plasmid could be horizontally transferred during this competition experiment and therefore increasing transconjugant populations must have resulted from clonal growth. Allowing for horizontal plasmid transfer (7-day conjugation experiment), however, the transconjugant frequency of RE2 and RE3 carrying p8A_IncF increased from the detection limit of 10^−6^ up to final frequencies of 1% (e.g. a 10^4^-fold increase in relative transconjugant population size; Fig. 4A,B). This large difference in transconjugant population increase with and without conjugation allows us to conclude that in our gut colonisation model without antibiotic selection, the spread of ESBL-plasmids was driven mainly by conjugative transfer, rather than by clonal expansion of transconjugants. The fast increase of the transconjugant population RE3 carrying p4A_IncI within only eight hours (Supplementary Fig. S15), despite a lack of growth advantage over recipient RE3 (Fig. 5C), further supports this result. However, we cannot exclude that clonal expansion did not contribute to observed final transconjugant frequencies. In fact, we expect that these are the result of both processes. For future work it might be interesting to systematically assess if such plasmid-mediated enhancement of host colonisation can contribute to the spread of some antibiotic resistance plasmids. Altogether, we demonstrated that variable plasmid spread probably resulted from variable transfer rates, that horizontal transfer allows for rapid ESBL-plasmid spread in the murine gut in the absence of antibiotic selection and that in vivo plasmid spread can reflect in vitro transfer dynamics.

### Plasmid transfer genes and incompatibility are the main genetic determinants of observed plasmid spread

We showed that horizontal transfer is a crucial determinant of the extent of ESBL-plasmid spread in vitro and in vivo and that in both systems, variability in final transconjugant frequencies across strain and plasmid combinations probably results from variable plasmid transfer rates. To explain the variability in these transfer rates, we analysed genetic factors of plasmids and donor and recipient strains that have previously been described to independently influence plasmid transfer [11, 12, 21, 22, 13–20].

**Fig. 6:**
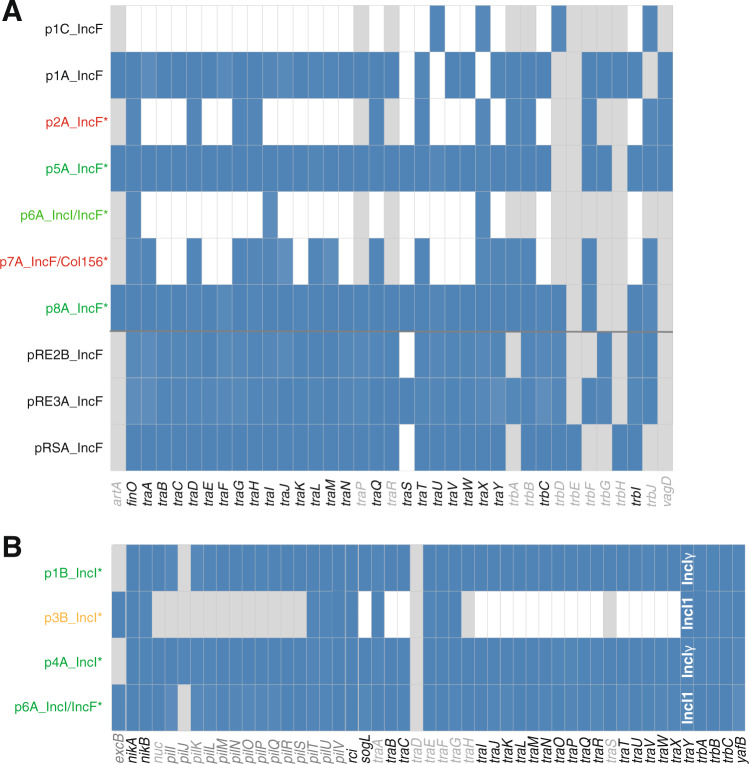
Transfer genes. Presence (blue) or absence (white) of essential tra genes for IncF plasmids (**A**), and IncI plasmids (**B**). Genes and panels in grey are non-essential for pilus biogenesis, DNA transfer or conjugation, according to Koraiman [51] (**A**) and Komano [49, 50] (**B**). ESBL-plasmids are indicated by an asterisk: green indicates spread to multiple recipient populations, orange indicates conjugation with only one replicate population, and red indicates no transfer. Non-ESBL-plasmids are labelled in black. For the traY gene of the IncI plasmids, we indicate whether the gene found corresponds to the version carried by IncI1 or IncIγ plasmids (white text in **B**). Plasmid p6A_IncI/IncF is shown on both **A** and **B**, but only the IncI1 transfer system is complete.

We found that only three out of the eight clinically relevant ESBL-plasmids encoded all necessary *tra* genes to initiate their own transfer (Fig. 6), suggesting only these plasmids would be conjugative. Consistent with this, each of these plasmids spread in at least three of the five recipients, whereas the other plasmids did not. This confirms the presence of essential *tra* genes on ESBL-plasmids to be the main genomic factor predicting their spread and the specific recipient strain to play a lesser role (Fig. 2). However, also p3B_IncI, which lacks most of the essential transfer genes, was transferred to RE2 in one of the six replicate populations. Although this occurred in only one replicate, this observation could warrant further work to see if non-conjugative ESBL-plasmids can be efficiently mobilised by conjugative plasmids present in donor or recipient strains (Supplementary Table S1).

Because our statistical analyses of the variation in plasmid spread after excluding non-conjugative plasmids indicated a significant additional plasmid effect and contributions of donor and recipient strains, we investigated the genetic basis of this more fine-scale variation in plasmid spread (Figs. 2, 3). The relatedness of plasmids in donor and recipient strains with regard to their replicons was a crucial determinant of the extent of plasmid spread. Plasmid incompatibility likely explains why final transconjugant frequencies of the IncFII ESBL-plasmids p5A_IncF and p6A_IncI/F were highest with recipient RE1, the only recipient without a plasmid encoding an IncFII-replicon, and varied largely depending on recipients (Fig. 2). The lack of transconjugants resulting from conjugation of donor D6 carrying plasmid p6A_IncI/F with recipient RS, could be due to the incompatibility with the resident plasmid pRS_IncF. Further evidence for the role of incompatibility comes from conjugation with plasmid p8A_IncF, which carries half of the IncFIC-FII replicon (Supplementary Fig. S7) and resulted in the loss of the resident F-plasmid pRE3A_IncF in recipient RE3, both in vitro and in vivo (Supplementary Table S2). Despite this plasmid interference in conjugation with RE3, plasmid p8A_IncF spread in all recipients at the same rate (Fig. 2).

Additionally, we have investigated the role of (i) phylogenetic relatedness of the mating strains, (ii) immunity systems such as RM and CRISPR-Cas systems, (iii) plasmid co-transfer and (iv) mutational changes accumulating during in vitro or in vivo conjugation assays in plasmid spread (see Supplementary Results). Of all these factors only plasmid transfer genes and plasmid incompatibility correlated with a detectable effect on plasmid spread in our in vitro and in vivo experiments.

## Discussion

We demonstrated that in addition to the transferring plasmid itself, strain aspects like the presence of plasmids in the recipient, are also important determinants of plasmid spread in the absence of antibiotic selection. This is consistent with and extends past work [2, 25, 27, 34] by quantifying the relative importance of donor, recipient and plasmid for the spread of key clinical plasmids from their native bacterial hosts. Moreover, the qualitative agreement between plasmid spread in vitro and in our mouse model demonstrated that these relative contributions are robust even in complex environments, suggesting in vitro screening can enable predictions of which plasmid–bacteria combinations will be most successful in nature. A second key implication of our results is that all of the ESBL-plasmids we tested that carried genes known to encode conjugative transfer machinery spread efficiently in various recipient populations, both in vitro and in vivo without antibiotic selection (Figs. 2–4). This shows predictions about the extent of plasmid spread in the absence of antibiotics can be further improved using sequence data annotated with information about conjugative machinery.

Despite the agreement between our in vivo and in vitro conditions, we stress that local abiotic conditions play a crucial role for conjugation and for plasmid spread. For instance, some plasmids can only transfer in a structured environment, as in surface mating, while other plasmids transfer at higher rates in well-mixed environments, as in liquid mating cultures. This phenomenon has been linked to pili flexibility [30, 31]. Whether the conjugative environment of the mammalian gut lumen resembles more a structured or a well-mixed environment is currently not clear. In the colonisation model we used for our conjugation experiments [58], interactions of bacteria with the host intestinal lining might allow for more structured populations [85]. Observed dynamics of plasmid p4_IncI indeed highlights a potential importance of structured environment for conjugation in vivo (Fig. 4). In combination with recipient RE3, plasmid p4A_IncI spread remarkably fast, reaching a transconjugant frequency of 1% already after 8 h (Supplementary Fig. S15). This transconjugant frequency was more than 100-fold higher compared to when p4A_IncI spread in recipient population RE2, a difference consistent with our in vitro surface mating experiment (Supplementary Fig. S12). This recipient-dependent difference further highlights the importance of host-encoded factors that can potentially influence plasmid spread in vivo. We speculate that such a factor might be the gene *iha* carried by RE3, which encodes an adherence-conferring molecule [86] and could influence its spatial organisation in the gut (Supplementary Fig. S6). Another indication for a fixed spatial structure of RE3, in fact of a spatially heterogenous structure of the involved bacterial populations, is the plateau the transconjugants of RE3 with p4A_IncI reach after 24 h [87]. Indeed, spatially distinct niches in the gut can exist and lead to differential growth or survival of *E. coli* strains [88]. Alternative explanations for this could be the out-competition of recipient and transconjugant populations by the plasmid donor strain, through either a direct interaction or an indirect ecological effect via interaction with microbiota members, or a reduced growth rate after initial colonisation of the plasmid donor strain. The waning population size of the recipients (Supplementary Fig. S14) may indeed influence the transconjugant population, and could be facilitated by T6SS-mediated killing (as RE3 does not contain a T6SS). This, however, seems unlikely as the sole cause of plasmid spread dynamics in RE3, because we would expect this to lead to a similar pattern with plasmid p8A_IncF from donor D8. We propose that surface mating could play an important role for plasmid spread in vivo and emphasise the need for future studies addressing this.

Based on bioinformatic analysis we investigated a potential role of further strain-specific features on plasmid spread. Bacterial defence systems can affect the efficiency of plasmid transfer [19] and all four recipient strains encode the adaptive immunity system CRISPR-Cas Type I, of which Type IF (recipients RE1 and RE2) is commonly associated with antimicrobial susceptibility in *E. coli* [89] (Supplementary Table S4). In laboratory *E. coli* strains such as K12, Type I CRISPR-Cas loci are considered to be inactive under laboratory growth conditions [90]. This is consistent with the observed lack of spacer acquisition in the laboratory for our natural strains. Further, it has been proposed that plasmid transfer to close kin is more efficient due to the similarity in RM systems of donor and recipient strains [28]. Here, based on presence and absence of RM systems, we did not find this relation. Our ESBL-plasmids, like many other conjugative plasmids [91], employ anti-restriction strategies and thus, we and others [92, 93] suggest that RM systems may only marginally shape horizontal plasmid transfer in natural systems, although to fully address this question a different experimental design would be required, using simpler conjugation assays and a much larger number of strains.

Our results also imply that interaction of plasmids present in donor and recipient strains can play a key role in their spread. This is important because in natural systems bacteria often harbour multiple plasmids. We found plasmid incompatibility to limit but not completely prevent plasmid transfer in conjugation with ST131 strains D5 and D6 (Fig. 2). This permeability probably results from the multiple replicons encoded on these ESBL-plasmids, a mechanism that could allow plasmids to transfer in spite of incompatibility [94]. Co-transfer of other plasmids is common, and has been proposed to affect plasmid transfer rates [14, 15, 95, 96]. We found little plasmid co-transfer and could not relate its occurrence to the observed plasmid spread (Supplementary Results and Table S2). Sequencing of the ESBL-plasmids in transconjugants revealed no mutations after transfer (Supplementary Results and Supplementary Table S2). This is in agreement with earlier findings reporting the absence of mutations on ESBL-plasmids even after 112 days of evolution of transconjugants [97] but does not exclude that such adaptive processes have happened in the past. Regardless, these data suggest that contemporary clinical ESBL-plasmids are well adapted to Enterobacteriaceae and do not require clone-specific adaptations for successful spread.

Given the central role of plasmids in the global dissemination of antibiotic resistance, it is of great importance to understand the factors contributing to plasmid spread under natural conditions. We demonstrated that in addition to the plasmid, strain aspects are key for plasmid spread in a complex mouse model. Crucially, we demonstrated this with natural, clinically relevant plasmid–strain combinations. Moreover, our study suggests large-scale in vitro conjugation experiments and genetic data, particularly annotated with information about plasmid conjugation machinery, can enable predictions about which plasmids will spread most rapidly and in which host strains in the gut environment of animals and humans. Ultimately, along with others [8], we advocate that early detection of successful strain–plasmid associations may allow for interventions that impede the emergence of pandemic strain–plasmid associations.

## Supplementary information

Supplementary Material

Supplementary Table S1

Supplementary Table S2
