# Supplementary material for: Plasmid- and strain-specific factors drive variation in ESBL-plasmid spread in vitro and in vivo
Source: ISME J. 2020 Nov 4;15(3):862–78. doi: 10.1038/s41396-020-00819-4 (PMC8026971; doi:10.1038/s41396-020-00819-4)
Supplement: Supplementary file 1 — Supplementary Material [file 41396_2020_819_MOESM1_ESM.docx]

**Supplementary Figures, Tables and Results**

**Figure S1 Plasmid transfer from native versus secondary host.** We marked D1 with the same Cm-resistance plasmid (pACYC184) as the *E. coli* recipients in the 1^st^ generation *in vitro* experiment, to exclude pACYC184 having a major effect on transconjugants’ ability to donate plasmids. Indeed, pACYC184 in D1 had a significantly negative effect on transfer of p1B_IncI to RE1 (Wilcoxon Rank Sum Test, *P* = 0.035 after Holm’s correction for multiple testing) and to RS (Student's t-Test, *P* = 0.042 after Holm’s correction for multiple testing). Although significant, the effect of pACYC184 was small compared to the difference in transconjugant frequency that resulted from transfer from native versus 2^nd^ generation donor strain. Circles represent independent replicates (*n = 5-6*) and the beams are mean values ± SEM. The detection limit was at ~10^-8^.

**Figure S2**. Control for transconjugant growth rates. To verify that the growth rate differences of recipients and transconjugants (Figure 5A-B) were not affected by the additional culturing steps transconjugants but not recipients have experienced in the 1^st^ generation conjugation experiment, we exposed recipients (RE1-RE3, *n = 4*) to the same additional culturing steps a conjugation assay would (black circles) and found no difference in growth rate to that of wild-type recipients (grey circles, see Materials and methods). Beams are mean values ± SEM.

**Figure S3 Maximal growth rates of donors and recipients used in the 1^st^ generation *in vitro* experiment.** Growth rates were estimated using OD measurements over 24 hours (*n=5*) in the absence of antibiotics.

**Figure S4 . Resistance genes.** Presence (blue) or absence (white) of resistance genes in donors (D1-D8) and recipients (RE1-RE3, RS). The quality score *q* reflects the percent identity *p* and coverage *c* of the BLAST match to the listed genes, where $q= \frac{p}{100}\cdot\frac{c}{100}$. The genes ant 3’’-Ia and ARR-3 were excluded from Supplementary Table S1. Chromosomal resistance genes are indicated with the red abbreviation " chromo ".

**Figure S5 Bacterial immunity systems.** Presence (blue) or absence (white) of CRISPR-Cas and restriction-modification systems (RM) in donors (D1-D8) and recipients (RE1-RE3, RS). CRISPR-Cas and RM systems were determined using prokka and confirmed using Rebase.

**Figure S6 Type 6 secretion systems and virulence genes.** Presence (blue) or absence (white) of Type 6 secretion systems (T6SS, defined by the presence of more than one prodigal T6SS-encoding gene in an operon) and virulence genes (found by BLAST) in donors (D1-D8) and recipients (RE1-RE3, RS). The quality score *q* reflects the percent identity *p* and coverage *c* of the BLAST match to the listed genes, where $q= \frac{p}{100}\cdot\frac{c}{100}$. Plasmid-based sequences are flagged with a red triangle. The only strain with a pathogenic virulence profile is D4, which carries chromosomally encoded intimin genes (*eae*, *tir*) commonly associated with Enteropathogenic *E. coli*. All strains except RE3 and D1, encode for a T6SS.

**Figure S7 Plasmid replicons.** Presence (blue) or absence (white) of plasmid replicons in donors (D1-D8) and recipients (RE1-RE3, RS). A red dot indicates the ESBL-plasmid, which can carry multiple replicons. The quality score *q* reflects the percent identity *p* and coverage *c* of the BLAST match to the listed genes, where $q= \frac{p}{100}\cdot\frac{c}{100}$. Three matches listed here, namely of p1C_IncF to IncFIC FII, p3A_crypt to IncFIB pKPHS1 (labelled “crypt”) and p8A_IncF to IncFIC FII were too short (< 75% coverage) to be included into Supplementary Table S1. IncY and p0111 are P1-like phages (indicated with the red label “phage”). The pESA2 replicon was found embedded in the chromosome (labelled “chromo”).

**Figure S8 Phages.** Presence (blue) and absence (white) of (pro)phages in donors (D1-D8) and recipients (RE1-RE3, RS). Plasmid-like phage sequences are flagged with a red triangle and the replicon of the contig they were found on (in white). All strains contain extensive (pro)phage related sequences.

**Figure S9 Mauve alignment.** This alignment shows that the p0111 “plasmids” of D2, D5, D8, and the IncY “plasmid” of RE2 are highly related to each other and to the phages SJ46 and P1. Although highly similar to the phage SSU5, the p3A_crypt “plasmid” of D3 was largely unrelated to the P1-like phages.

**Figure S10 Toxin/Antitoxin-systems.** Presence (blue) or absence (white) of Toxin/Antitoxin-systems (TA) in donors (D1-D8) and recipients (RE1-RE3, RS). Brackets indicate T/AT-pairs. The ESBL-plasmids are indicated by an asterisk. IncI-plasmids and IncF-plasmids are generally associated with distinct TA-systems.

**Figure S11 Absolute size of donor, recipient, and transconjugant populations in the 1^st^ generation *in vitro* experiment.** We performed conjugation experiments with natural donor-plasmid pairs and recipients RE1 (A), RE2 (B), RE3 (C) and RS (D). Beams are mean values ± SEM (*n = 6)*, dotted lines indicate the detection limit by selective plating, grey for donors and recipients, black for transconjugants.

**Figure S12** **Surface-mating experiment.** We allowed donor strains D1, D4, D6 and D8 to conjugate with all four recipients (colours; legend) on LB-agar plates (*n = 4*). Resulting transconjugant frequencies depended on donor-plasmid pair and the recipient strain. Beams are mean values ± SEM, the dotted line indicates the detection limit by selective plating.

**Figure S13 Absolute size of donor, recipient, and transconjugant populations in the 2^nd^ generation *in vitro* experiment.** We performed conjugation experiments with transconjugants from the 1^st^ generation *in vitro* experiment as plasmid donors and recipients RE1 (A), RE2 (B,) and RS (C). Beams are mean values ± SEM (*n = 6*), dotted lines indicate the detection limit by selective plating, grey for donors and recipients, black for transconjugants.

**Figure S14 Total populations *in vivo*.** Each panel reflects one donor-recipient pair and represents the population sizes of donors, recipients, and transconjugants in Fig. 4. A) Conjugation from D4 to RE2 (*n = 7*). B) Conjugation from D4 to RE3 (*n = 7*). C) Conjugation from D8 to RE2 ( *n= 7*). D) Conjugation from D8 to RE3 (*n = 10*). E) Conjugation from D7 to RE2 (*n = 7*). F) Conjugation from D7 to RE3 (*n = 10*). The dotted line indicates the detection line by selective plating. The solid lines indicate the median.

**Figure S15 Rapid spread of p4A_IncI with recipient RE3.** We monitored plasmid spread within the first 24 hours post donor infection. Plasmid spread is reported as final transconjugant frequency and enumerated in faeces by selective plating. The solid line indicates the median (*n = 5*), the dotted line indicate the detection limit for selective plating.

**Figure S16 Direct competition between donors and recipients *in vivo.*** We performed competition experiments by colonizing the mice with a 1:1 mix of plasmid donors with RE2 (A) (*n=7*) or RE3 (B) (*n=7* for D4-RE3; *n=10* for D8-RE3 and D7-RE3). The relative frequency was calculated by dividing the recipient population by the donor strain population.

**Supplementary Tables**

**Table S1** Strain overview. Table S1 is a separate file. Overview of all strains used in this study, including their sequence type (ST), natural plasmid content and detected resistance genes. Replicon and resistance gene hits are only shown in this table if they had a coverage and percent identity of at least 70%, leading to a few differences with respect to Figures S4 and S7, most notably IncFIC_FII in D8. Contiguous sequences (contigs) were denoted “c” or “p” for chromosomal or plasmid, respectively. Short sequences (up to 5 kB) that mapped to the own chromosome, and any contigs smaller than 1kB were removed. Any remaining contigs without a known replication gene were denoted “_crypt” for cryptic.

**Table S2 Overview of plasmid transferring and mutations accumulating during conjugation experiments.** Table S2 is a separate file.

**Table S3 Priors for the phylogenetic tree inference.** Parameter priors for the birth-death tree prior.

| Become Uninfectious Rate | LogNormal (mu = 0, sigma = 1) |
| --- | --- |
| Clock rate | LogNormal (mu = 0.4, sigma = 1) |
| Kappa | LogNormal (mu = 1.5, sigma = 1.25) |
| Origin | LogNormal (mu = 6, sigma = 1.25) |
| Reproductive Number | LogNormal (mu = 0, sigma = 1) |
| Sampling Proportion | Beta (alpha = 1.2, beta = 10) |

**Table S4 Phenotypic resistance profile of donor strains.** Minimum inhibitory concentration (µg/mL) measurements of ESBL donors used in this study. The ESBL-resistance phenotype was defined by resistance to Ceftriaxone and Ceftazidime.

| **Strain** | **Amp** | **Amo/C** | **Pip-T** | **Ceftaz** | **Ceftri** | **Cefe** | **Erta** | **Imi** | **Mero** | **Tobra** | **Amika** | **Cotri** | **Cipro** | **Coli** |
| --- | --- | --- | --- | --- | --- | --- | --- | --- | --- | --- | --- | --- | --- | --- |
| **D1** | >=32 | 4 | <=4 | <=1 | 32 | 2 | <=0.5 | <=0.25 | <=0.25 | <=1 | <=2 | >=320 | <=0.25 | <=0.5 |
| **D2** | >=32 | >=32 | 8 | 4 | 32 | 2 | <=0.5 | <=0.25 | <=0.25 | >=16 | 4 | >=320 | >=4 | <=0.5 |
| **D3** | >=32 | >=32 | >=128 | >=64 | >=64 | >=64 | <=0.5 | <=0.25 | <=0.25 | >=16 | >=64 | >=320 | >=4 | <=0.5 |
| **D4** | >=32 | 8 | <=4 | <=1 | >=64 | <=1 | <=0.5 | <=0.25 | <=0.25 | <=1 | <=2 | <=20 | <=0.5 | <=0.5 |
| **D5** | >=32 | 16 | 8 | 16 | >=64 | >=64 | <=0.5 | <=0.25 | <=0.25 | >=16 | 16 | >=320 | >=4 | <=0.5 |
| **D6** | >=32 | 4 | <=4 | <=1 | >=64 | >=64 | <=0.5 | <=0.25 | <=0.25 | 8 | <=2 | <=20 | >=4 | <=0.5 |
| **D7** | >=32 | 4 | <=4 | 4 | >=64 | 4 | <=0.5 | <=0.25 | <=0.25 | <=1 | <=2 | >=320 | >=4 | <=0.5 |
| **D8** | >=32 | 4 | 4 | <=1 | >=64 | <=1 | <=0.5 | <=0.25 | <=0.25 | <=1 | <=2 | >=320 | <=0.25 | <=0.5 |
| Abbreviations: **Amp** = Ampicillin; **Amo/C** = Amoxicillin/Clavulanic acid; **Pip-T** = Piperacillin-Tazobactam; **Ceftaz** = Ceftazidime; **Ceftri** = Ceftriaxone; **Cefe** = Cefepim; **Erta** = Ertapenem; **Imi** = Imipenem; **Mero** = Meropenem; **Tobra** = Tobramycin; **Amik**a = Amikacin; **Cotri** = Cotrimoxazol; **Cipro** = Ciprofloxacin; **Coli** = Colistin. Interpretatin of MIC breakpoints according to EUCAST guidelines (v8.1). | | | | | | | | | | | | | | |

**Supplementary Results**

**Importance of clonal expansion of transconjugants for observed plasmid spread**

To determine the importance of clonal expansion of transconjugants for observed plasmid spread, we calculated whether the final transconjugant frequencies of 10 plasmid-strain combinations (excluding RE2 and RE3 carrying p1B_IncI due to missing data) could result from clonal expansion of transconjugants after a single initial plasmid transfer to a recipient cell (1^st^ generation *in vitro* experiment, Figure 2). That is, we asked whether appreciable horizontal transfer rates are required to explain our results.

Using the observed recipient growth rates, we calculated the transconjugant growth rates that would be needed to explain observed final transconjugant frequencies from clonal expansion following a single transfer event at the start of the assay (see tables below). To simplify the calculation, we assumed exponential growth of the recipient and transconjugant population, starting from *R*(0) and a single individual *T*(0)=1 respectively.

That is, the final transconjugant frequency (*f*) at a time *t* is determined as follows:

$$f(t)=\frac{T(t)}{R\left( t \right)+T(t)}=\frac{T(0)e^{\psi_{T}t}}{R(0)e^{\psi_{R}t}+T(0)e^{\psi_{T}t}}$$

where we have assumed simple exponential growth for the second equality, and ψ_R,_ ψ_T_ describe the recipient and transconjugant growth rate, respectively.

Using these assumptions, one can calculate the minimal transconjugant growth rate ψ_T_ needed to explain the final transconjugant frequency after e.g. 24 hours:

$$\begin{aligned} \psi_{T}>\psi_{R}-\frac{1}{24}\ln\left( \frac{\frac{1}{f}-1}{R\left( 0 \right)} \right) \#\left( 1 \right) \end{aligned}$$

For two strain-plasmid pairs (transconjugant RS carrying p1B_IncI and p8A_IncF), the measured growth rate of transconjugants was greater than the calculated minimal growth rate needed to explain their observed final transconjugant frequencies. Thus, for these two strain-plasmid pairs, final transconjugant frequencies could have been reached predominantly by clonal expansion of transconjugants after only a small number of transfer events, without requiring appreciable numbers of independent horizontal plasmid transfer events. Our calculations, however, are very conservative, because of the assumption of 24 hours of exponential growth to reach observed transconjugant frequencies. In reality, our bacterial cultures had much shorter exponential growth phases, followed by stationary growth. Therefore, our calculations underestimate the minimal transconjugant growth rates that would be required to explain the observed final transconjugant frequencies from clonal expansion.

| **Measured populations growth rates** of recipients and transconjugants. The data is the same as in the plasmid cost comparison (Figure 5A/B) and stems from manual OD measurements over 24 hours (*n = 12*). Note that these absolute growth rates differ from the ones estimates for Figures S2 and S3 (See Materials and methods).   \|  \| RE1_pACYC184 \| RE2_pACYC184 \| RE3_pACYC184 \| RS_marTcat \| \| --- \| --- \| --- \| --- \| --- \| \| - (recipient) \| 0.96 ± 0.01 \| 0.98 ± 0.01 \| 0.66 ± 0.006 \| 0.34 ± 0.002 \| \| with p1B_IncI \| 0.99 ± 0.01 \| - \| - \| **0.38** ± 0.009 \| \| with p4A_IncI \| 0.95 ± 0.01 \| 0.95 ± 0.01 \| 0.70 ± 0.006 \| 0.37 ± 0.001 \| \| with p8A_IncF \| 0.88 ± 0.004 \| 0.99 ± 0.006 \| 0.67 ± 0.004 \| **0.38** ± 0.0003 \|   **Calculated minimal transconjugant growth rates** needed to explain the observed final transconjugant frequencies (Figure 2).   \|  \| RE1_pACYC184 \| RE2_pACYC184 \| RE3_pACYC184 \| RS_marTcat \| \| --- \| --- \| --- \| --- \| --- \| \| with p1B_IncI \| 1.07 \| 1.06 \| 0.75 \| **0.36** \| \| with p4A_IncI \| 1.23 \| 1.16 \| 0.96 \| 0.60 \| \| with p8A_IncF \| 1.10 \| 1.13 \| 0.80 \| **0.36** \| |
| --- | --- | --- | --- | --- | --- | --- | --- | --- | --- | --- | --- | --- | --- | --- | --- | --- | --- | --- | --- | --- | --- | --- | --- | --- | --- | --- | --- | --- | --- | --- | --- | --- | --- | --- | --- | --- | --- | --- | --- | --- | --- | --- | --- | --- | --- |

**Genomic factors potentially affecting plasmid transfer rates**

i) the phylogenetic relatedness of the mating strains

In contrast, to the relatedness of plasmids, the phylogenetic relatedness of the mating strains (Figure 1) could not explain the observed plasmid transfer dynamics: donors D5 and D6 are equally closely related to recipients RE1 and RE2, yet we observed very different final transconjugants frequencies (Figure 2). Additionally, the Salmonella recipient RS is phylogenetically distant to all donors, yet the variation in final transconjugants frequency was similar to that observed across *E. coli* recipients.

ii) immunity systems such as RM and CRISPR-Cas systems

We found various RM systems (Supplementary Figure S5) and investigated whether ESBL-plasmid transfer from an RM deficient donor into a recipient with this RM system could explain reduced plasmid transfer. We found high levels of self-self transfer (Figure 3), which could result from identical RM systems in donor and recipient strains but for other mating pairs, we found no relation between presence/ absence of RM systems and transfer. The anti-restriction protein YfjX (ardB) is present in nearly all strains, including on all ESBL-plasmids, and could reduce the detrimental effect of RM systems in recipients. We investigated whether the adaptive immunity systems CRISPR-Cas Type 1F (recipients RE1 and RE2) and CRISPR-Cas Type 1E (recipients RE3, RS and donors D1-4, D8) could have been a barrier to conjugation. Screening the spacer sequences in recipient strains did not reveal any matches with any of our plasmid or phage sequences and neither *in vitro* nor *in vivo* have the CRISPR arrays in transconjugants acquired any new spacers compared to the recipients.

iii) plasmid co-transfer

Our donor and recipient strains carry multiple non-ESBL plasmids whose co-transfer could have affected ESBL-plasmid transfer rates. However, sequencing revealed only a few isolated cases of co-transferring plasmids *in vitro* and none *in vivo*. For plasmid p8C_IncBOKZ we found some transfer to all four recipients of the 1^st^ generation experiment and from there to further recipients in the 2^nd^ generation experiment. This process seemed independent of donor and recipient strains (Supplementary Table S2). The only small Col-plasmids that were transferred were p1D_ColRNAI and p8G_Col8282. The P1 phage-like plasmid p8B_p0111 transferred *in vitro* to RE3 but not *in vivo* (Supplementary Table S2). Although they encode the *tra* genes (S2 File), the resident plasmids in the recipients (RE2, RE3 and RS) showed no horizontal transfer.

iv) mutational changes accumulating during *in vitro* or *in vivo* conjugation assays

Sequencing transconjugants (Supplementary Table S2) allowed us to screen for mutations that could explain some of the variation in final transconjugant frequencies across donor-recipient pairs or between primary and secondary plasmid transfer (Figures 2-3). We found no mutations on ESBL-plasmids, neither after the 24-hours *in vitro* nor the 7-days *in vivo* conjugation experiments (Supplementary Table S2). The only consistent changes on plasmids were the mutations of P1 phage-like plasmid p8B_p0111, which was passed to recipient RE3 *in vitro,* but not *in vivo*: here we found mutations in the genomic region encoding side tail fibre proteins. Similar mutations were also frequently present within chromosomal prophages. We found several other chromosomal mutations in transconjugants (Supplementary Table S2). For instance, all transconjugants resulting from conjugation into RE1 recipients (*n = 11*) and some (*n = 9* out of 22) RE3 recipients showed intergenic mutations in the promoter sequence or the phase ON/OFF region of the *fim* operon. The *fim* genes encode the type 1 pilus (type 1 fimbria), a virulence factor responsible for cell adherence (1). RE1 chromosomally encodes for two mobilization proteins mobA, required for the mobilization of plasmid and conjugative transposons. In all 11 sequenced RE1-transconjugants, we found various intergenic mutations upstream of at least one *mobA*.

1. Schwan WR. Regulation of fim genes in uropathogenic Escherichia coli. World J Clin Infect Dis. 2011;1(1):17.
