# Supplementary material for: Plasmid- and strain-specific factors drive variation in ESBL-plasmid spread in vitro and in vivo
Source: ISME J. 2020 Nov 4;15(3):862–78. doi: 10.1038/s41396-020-00819-4 (PMC8026971; doi:10.1038/s41396-020-00819-4)
Supplement: Supplementary file 2 — Supplementary Table S1 [file 41396_2020_819_MOESM2_ESM.docx]

### **D1 (ST117)**

| **Contig** | **Size** | **Replicon** | **Resistance Genes** |
| --- | --- | --- | --- |
| c1 | (not closed) | - Chromosome - | mdf(A) |
| p1A_IncF | 153.9 kB | IncFIA; IncFIB; IncFIC(FII) | - |
| p1B_IncI | 111 kB | IncIγ | aadA5; blaCTX-M-1; dfrA17; sul2 |
| p1C_IncF | 86 kB | - | - |
| p1D_ColRNAI | 7 kB | ColRNAI | - |

## **D2 (ST648)**

| **Contig** | **Size** | **Replicon** | **Resistance Genes** |
| --- | --- | --- | --- |
| c2 | 5.2 Mbp | - Chromosome - | mdf(A); tet(34) |
| p2A_IncF | 165.7 kB | IncFIA; IncFIB; IncFII | mph(A); catB3; aadA5; aac(6')-Ib-cr; dfrA17; sul1; sul2; blaOXA-1; tet(A); blaCTX-M-27; aph(3'')-Ib; erm(B) |
| p2B_p0111 | 97.8 kB | p0111 (phage) | - |
| p2C_Col156 | 5.2 kB | Col156 | - |
| p2D_Col156 | 5.2 kB | Col156 | - |
| p2E_Col8282 | 4.1 kB | Col8282 | - |
| p2F_ColMG8282 | 1.5 kB | Col(MG8282) | - |

## **D3 (ST 648)**

| **Contig** | **Size** | **Replicon** | **Resistance Genes** |
| --- | --- | --- | --- |
| c3 | 5.0 MBp | - Chromosome - | mdf(A); tet(34) |
| p3A_crypt | 109.5 kB | Similar to phage SSU5 | - |
| p3B_IncI | 59.8 kB | IncI1 | blaCMY-42 |
| p3C_crypt | 4.1 kB | - | - |

## **D4 (ST40)**

| **Contig** | **Size** | **Replicon** | **Resistance Genes** |
| --- | --- | --- | --- |
| c4 | 5.0 MBp | - Chromosome - | mdf(A); tet(34) |
| p4A_IncI | 88.9 kB | IncIγ | blaCTX-M-1 |
| p4B_ColRNAI | 5.3 kB | Col(MGD2); ColRNAI | - |
| p4C_Col156 | 4.7 kB | Col156 | - |

## **D5 (ST131)**

| **Contig** | **Size** | **Replicon** | **Resistance Genes** |
| --- | --- | --- | --- |
| c5 | (not closed) | - Chromosome - | mdf(A) |
| p5A_IncF | 160 kB | IncFIB; IncFIC(FII); IncFIA | mph(A); aadA5; aac(6')-Ib-cr; dfrA17; sul1; blaCTX-M-15; aac(3)-IIa; tet(A); catB3; blaOXA-1 |
| p5B_p0111 | 91.3 kB | p0111 (phage) | - |
| p5C_ColBS512 | 2 kB | Col(BS512) | - |

## **D6 (ST131)**

| **Contig** | **Size** | **Replicon** | **Resistance Genes** |
| --- | --- | --- | --- |
| c6 | 4.96 MBp | - Chromosome - | mdf(A) |
| p6A_IncI/F | 157 kB | IncI1; IncFIA; IncFIB; IncFII | blaTEM-1B, aac(3)-IId, blaCTX-M-8 |
| p6B_IncX4 | 33.1 kB | IncX4 | - |
| p6C_crypt | 29.1 kB | - | - |
| p6D_ ColRNAI | 8.6 kB | ColRNAI | - |
| p6E_Col156 | 6.2 kB | Col156 | - |
| p6F_Col156 | 5.2 kB | Col156 | - |
| p6G_ColKPHS6 | 2.6 kB | Col(KPHS6) | - |

## **D7 (ST131)**

| **Contig** | **Size** | **Replicon** | **Resistance Genes** |
| --- | --- | --- | --- |
| c7 | 4.97 MBp | - Chromosome - | mdf(A) |
| p7A_IncF | 134.9 kB | IncFIA; IncFIB; IncFII; Col156 | mph(A); aph(3'')-Ib; aadA5; dfrA17; sul1; sul2; tet(A); blaCTX-M-27 |
| p7B_Col156 | 5.2 kB | Col156 | - |

## **D8 (ST69)**

| **Contig** | **Size** | **Replicon** | **Resistance Genes** |
| --- | --- | --- | --- |
| c8 | 5.07 MBp | - Chromosome - | mdf(A) |
| p8A_IncF | 131 kB | IncFIA; IncFIB | dfrA14, mph(A), blaCTX-M-14, tet(B) |
| p8B_p0111 | 95.9 kB | p0111 (phage) | - |
| p8C_IncBOKZ | 93.5 kB | IncB/O/K/Z | - |
| p8D_ColRNAI | 7.9 kB | ColRNAI | - |
| p8E_Col156 | 7.5 kB | Col156 | - |
| p8F_ColRNAI | 5.6 kB | ColRNAI | - |
| p8G_Col8282 | 4.1 kB | Col8282 | - |
| p8H_ColMG8282 | 1.6 kB | Col(MG8282) | - |

## **RE1 (ST80)**

| **Contig** | **Size** | **Replicon** | **Resistance Genes** |
| --- | --- | --- | --- |
| cRE1 | 5.2 MBp | - Chromosome –  pESA2 | mdf(A) |
| pRE1A_ColRNAI | 4.6 kB | ColRNAI | - |

## **RE2 (ST95)**

| **Contig** | **Size** | **Replicon** | **Resistance Genes** |
| --- | --- | --- | --- |
| cRE2 | (not closed) | - Chromosome - | mdf(A), tet(34) |
| pRE2A_IncY | 94.6 kB | IncY (phage) | - |
| pRE2B_IncF | 72.9 kB | IncFII | - |
| pRE2C_crypt | 22.2 kB | (similar to phage SFII) | - |

## **RE3 (ST6697)**

| **Contig** | **Size** | **Replicon** | **Resistance Genes** |
| --- | --- | --- | --- |
| cRE3 | (not closed) | - Chromosome - | mdf(A), tet(34) |
| pRE3A_IncF | 93.5 kB | IncFIC(FII) | - |
| pRE3B_crypt | 77 kB |  | - |
| pRE3C_crypt | 2.5 kB | - | - |

## **RS (14028S)**

| **Contig** | **Size** | **Replicon** | **Resistance Genes** |
| --- | --- | --- | --- |
| cRS |  | - Chromosome - | aac(6’)-Iaa, mdf(A) |
| pRS_IncF |  | IncFII(S); IncFIB(S) | - |
